# The validity of spatiotemporal gait analysis using dual laser range sensors: a cross-sectional study

**DOI:** 10.1186/s40945-019-0055-6

**Published:** 2019-02-19

**Authors:** Masanobu Iwai, Soichiro Koyama, Shigeo Tanabe, Shohei Osawa, Kazuya Takeda, Ikuo Motoya, Hiroaki Sakurai, Yoshikiyo Kanada, Nobutoshi Kawamura

**Affiliations:** 1 Department of Rehabilitation, Kawamura Hospital, Gifu, 501-3144 Japan; 2 Faculty of Rehabilitation, School of Health Sciences, Fujita Health University, 98-1 Dengakugakubo, Kutsukake, Toyoake, Aichi 470-1192 Japan; 3 Department of Rehabilitation, Tajimidaiichi Hospital, Tajimi, 507-0007 Japan; 4 Department of Neurology, Kawamura Hospital, Gifu, 501-3144 Japan

**Keywords:** Gait analysis, Laser range sensor, Spatiotemporal analysis, Instrumented walkway

## Abstract

**Background:**

The spatiotemporal parameters were used for sophisticated gait analysis in widespread clinical use. Recently, a laser range sensor has been proposed as a new device for the spatiotemporal gait measurement. However, measurement using a single laser range sensor can only be used for short-range gait measurements because the device irradiates participants with lasers in a radial manner. For long-range gait measurement, the present study uses a modified method using dual laser range sensors installed at opposite ends of the walking path. The aim of present study was to investigate the concurrent validity of the proposed method for spatiotemporal gait measurement by comparison to a computer-based instrumented walkway system.

**Methods:**

Ten healthy participants were enrolled in this study. Ten-meter walking tests at 100, 75, and 50% of the comfortable speed were conducted to determine the concurrent validity of the proposed method compared to instrumented walkway measurements. Frequency distributions of errors for foot-contact (FC) and foot-off (FO) estimated times between the two systems were also calculated to determine the adequacy of estimation of FC and FO from three perspectives: accuracy (smallness of mean error), precision (smallness of variability), and unambiguity (monomodality of histogram). Intra-class correlation coefficient (2,1) was used to determine the concurrent validity of spatiotemporal parameters between the two systems.

**Result:**

The results indicate that the detection times for FC and FO estimated by the proposed method did not differ from those measured by the instrumented walkway reference system. In addition, histogram for FC and FO showed monomodality. Intra-class correlation coefficients of the spatiotemporal parameters (stance time: 0.74; double support time: 0.56; stride time: 0.89; stride length: 0.83; step length: 0.71; swing time: 0.23) were not high enough. The mean errors of all spatiotemporal parameters were small.

**Conclusions:**

These results suggest that the proposed lacks sufficient concurrent validity for spatiotemporal gait measurement. Further improvement of this proposed system seems necessary.

**Trial registration:**

UMIN000032710. Registered 24 May 2018. Retrospectively registered.

## Background

In gait disorder rehabilitation, gait analysis plays an important role in optimizing treatment for each patient [1–4]. Conventionally, visual observation of gait analysis is easy and low cost and is commonly used in rehabilitation facilities. However, previous studies report that visual observation gait analysis has low inter-rater and test-retest reliability as well as low criterion concurrent validity in contrast to kinematic analyses using various instruments [4, 5]. For highly accurate measurements with good inter-rater and test-retest reliability, a three-dimensional motion analysis system has been used. Although this system is able to measure whole-body joint motions, it has high costs and is time- and labor-intensive to set up [6].

Spatiotemporal gait measurement is another valuable method to identify gait deviations, make diagnoses, determine appropriate therapy, and monitor patient progress [2, 3]. Frequently, parameters such stance time, swing time, double support time, stride time, stride length, and step length are evaluated [7–10]. To calculate these spatiotemporal parameters, accurate detection of two events for switching between the stance and swing phases is essential: foot contact (FC) and foot off (FO). FC is defined as when any point of the foot first contacts and is the starting point of the stance phase. FO is when the sole is raised completely from the floor and is the onset of the swing phase. A measurement system for detection of FC and FO is a computer-based instrumented walkway system with pressure sensors and produces high inter-rater and test-retest reliability [2, 7–10]. Although this system has a relatively reasonable price as compared with a three-dimensional motion analysis system, it is still considerably expensive to become widely used. In addition, it occupies a large amount of floor space and greatly limits effective use of the exercise room. While this system is placed on the floor, the place is not able to be used for other purposes even though the exercise room has limited floor space.

Recently, spatiotemporal gait measurement using a laser range scanner has been proposed as easy to install and remove [11–14]. With a laser range scanner, both lower legs are measured using two best-fitting circles whose contours are defined by laser points. Although this method is useful for easy measurement of gait parameters in a clinical setting, the raw contour of the leg is incomplete because the sensor provides only one-sided information [11]. In addition, the number of laser points comprising the spheres decreases with long-range gait measurements because the lasers irradiate participants in a radial manner. Since the radial range decreases with increasing distance from the laser, this causes larger measurement errors.

For eliminating problems in long-range gait measurement, we proposed a method of spatiotemporal gait analysis using dual laser range sensors installed at opposite ends of the walking path. Because the measurement using laser range sensor is quick and easy method, this proposed method has a high degree of usability for clinical practice. However, it is not clear whether the proposed method has concurrent validity, which is defined as evaluation of an instrument against an already validated measure [15], for spatiotemporal gait measurement by comparison to a computer based instrumented walkway system (reference system) that was widely used for criterion-related validity. The aim of present study was to investigate the concurrent validity of the proposed method for spatiotemporal gait measurement by comparison to a reference system.

## Methods

### Participants

Ten healthy participants (7 males and 3 females, 20–24 years of age, 154-184 cm in height, 49-70 kg in weight) were enrolled in the present study. All participants have no history of orthopedic, neurophysiologic, and cardiovascular diseases. Informed consent was obtained from each participant before the experiments. The present study was approved by the ethics committee and was conducted according to the Declaration of Helsinki for human experiments.

### Experimental procedures

This study used a cross-sectional design to assess the concurrent validity of the proposed method for spatiotemporal gait measurement by comparison to a reference system.

Participants wearing short pants were asked to get on a walking path and walk barefoot along a 12 m straight line including 3.5 m in front of the measured walking path and 3.5 m beyond the end of walking path. Each participant performed one trial at each speed: 100, 75, and 50% of the comfortable speed in a subjective manner. Before measurement, the order of the speed conditions was randomized for each participant. During the gait test, spatiotemporal measurements were carried out simultaneously using both the proposed method and the reference system. The inter-trial interval was set to 2 minutes to prevent fatigue.

### Proposed method using laser range sensors

**Fig. 1:**
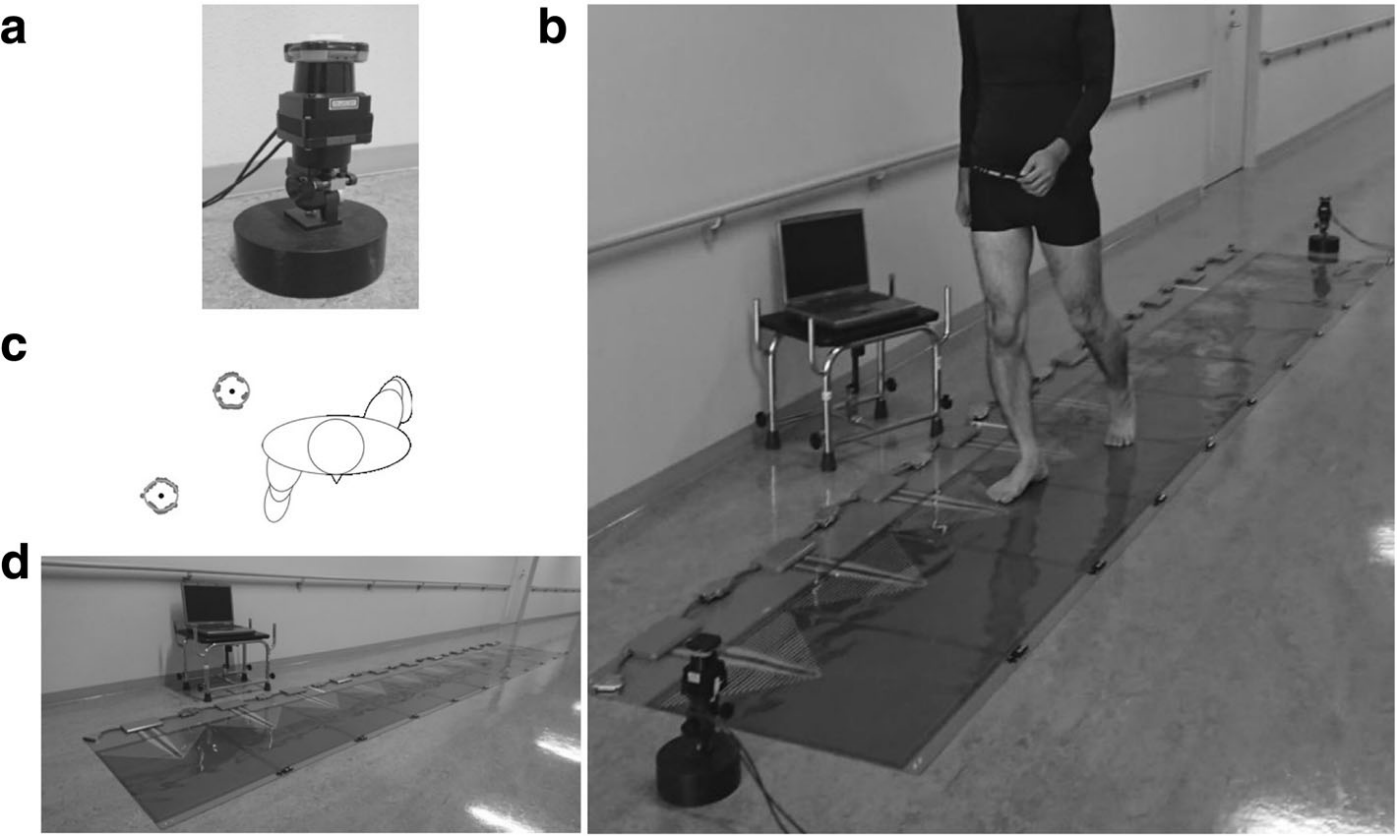
Experimental setup and measurement devices: **a**) A two-dimensional radial scanning laser range sensor (UTM-30LX, Hokuyo Automatic Co., Ltd., Osaka, Japan). **b**) The position and configuration of dual laser range sensors. **c**) Shin contour and hypothetical center of the leg calculated from the dual laser range sensors. **d**) A computer-based instrumented walkway system (Walk-way MG-1000, Anima, Japan)

A two-dimensional radial scanning laser range sensor (UTM-30LX, Hokuyo Automatic Co., Ltd., Osaka, Japan) was used (Fig. 1a). The device has a scanning range from − 135° to 135° in steps of 0.25° (total of 1080 data points measuring the distance from the sensor to the target), and one scan is completed in 0.025 s (i.e., the sampling frequency is 40 Hz). In addition, the device exhibits very small test-retest variability and the relative error of a distance (0.1 to 10 m, σ < 0.01 m and ± 0.01 m, white Kent paper, respectively) in the repeated measurements using same laser range sensor unit (i.e. unit testing). Two devices were installed at opposite ends of a five-meter walking path at the level of the average shin height (0.25 m above the floor) [16] (Fig. 1b).

By radiating from both sides, the round shape on each shin can be extracted. When walking is started, the front side laser acquires more data points than the back side. At the middle point, the number of data from both sides are almost the same. At the end of walking, the back side laser generates more data than the front laser. The data can be separated into anterior-posterior and medial-lateral distances using the distances from the sensor to the target and the angle of the laser using trigonometric functions. Each shin contour, consisting of several data points obtained from the laser range sensors, was extracted from the total data. The center of the best-fitting circle was defined as the hypothetical center of the leg (Fig. 1c). A moving average of 13 consecutive data points for each leg coordinate was calculated for smoothing the time series data. In addition, the data was converted into acceleration data to estimate the time of FC and FO. In the present study, FC time was defined as the minimum of the smoothed time series acceleration values, and FO time was defined when the smoothed time series acceleration values reached its maximum. FC and FO positions were defined as the distances from the proximal side laser at FC and FO times in the anterior-posterior direction.

In the present study, six spatiotemporal parameters (i.e., stance time, swing time, double support time, stride time, stride length and step length) were calculated. Stance time was defined as the time interval from ipsilateral FC-time to FO-time. Swing time was defined as the time interval from ipsilateral FO-time to FC-time. Double support time was defined as the time interval from contralateral FC-time to FO-time. The stride time was defined as the time interval between consecutive ipsilateral FC-times. Stride length was defined as the difference in consecutive ipsilateral FC-positions. Step length was defined as the difference in consecutive contralateral FC-positions. To acquire and analyze the data, LabVIEW software version 11.0.1 (National Instruments, Tokyo, Japan) was used.

### Reference system

A computer-based instrumented walkway system (Walk-way MG-1000, Anima, Japan) was used as the reference system (Fig. 1d). This reference system is able to determine the spatiotemporal parameters of gait from on/off signals between the participant’s foot and the surface of the sensors at a sampling frequency of 100 Hz. The length and width of the walkway are 4.8 m and 0.82 m, respectively. Although the validity and reliability of this reference system on the parameters used in this study have not been established yet, the concurrent validity and test-retest reliability of GAITRite system, which is similar instrument and widely used in gait analysis, have been already examined and established using inter- and intra-class correlation coefficient (> 0.84 and > 0.93, respectively) [10, 17]. The reference system was installed between dual laser sensors. In the walkway system, data were obtained and processed using software embedded in the system.

### Statistical analysis

The mean estimation error for each participant was calculated by subtracting the reference system data from the proposed experimental system data. A negative error represents a late laser estimation. Frequency distributions of errors for FC and FO times between the two systems were also calculated to determine the adequacy of estimation of FC and FO by the acceleration data from three perspectives: accuracy (smallness of mean error), precision (smallness of variability), and unambiguity (monomodality of histogram). The concurrent validity of six spatiotemporal parameters between the two systems were examined using the intra-class correlation coefficient (ICC(2,1)). The ICCs were calculated based on single measurement, absolute agreement, two-way random effects model according to previous studies [18]. In accordance with Portney et al.’s classification, “ICC > 0.75” was interpreted as good [19]. To demonstrate the spread of differences of the individual pairs in each parameter, Bland-Altman (BA) plots were used [20]. And then, to describe the absolute agreement between the proposed and reference systems, 95% limits of agreement (95% LoA) were used. The 95% LoA was calculated as ±1.96 standard deviation (SD) of the differences between both systems according to previous studies [20, 21]. In addition, paired t-test was used to exclude systematic error between both systems in each parameter. Statistical significance was set at *P* < 0.05. All statistical analysis was performed using IBM SPSS Statistics version 21 for Windows.

## Results

**Fig. 2:**
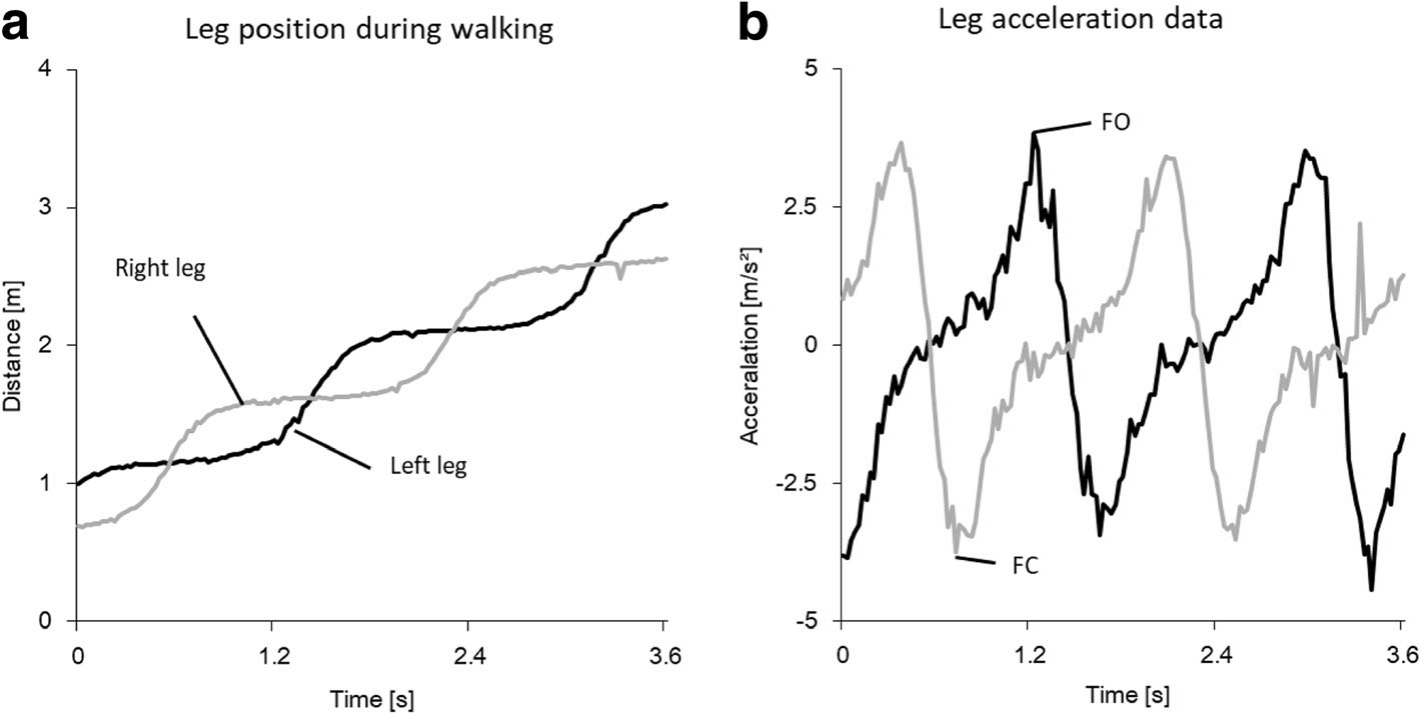
Typical time-course data of each leg position and acceleration calculated from laser range sensors: **a**) Example of each leg position during walking where the x-axis indicates time (s) and the y-axis indicates the distance from the proximal side laser to the participant’s shin (m). **b**) Example of each leg acceleration derived from position data. The y-axis indicates the acceleration values (m/s^2^). Foot contact (FC) time was defined as the minimum of the smoothed time series acceleration values, and foot off (FO) time was defined as the maximum of the smoothed time series acceleration values. The black and gray solid line indicate the data of each leg which are smoothed by the moving average. The black solid line indicates left leg and the gray solid line indicates right leg

**Fig. 3:**
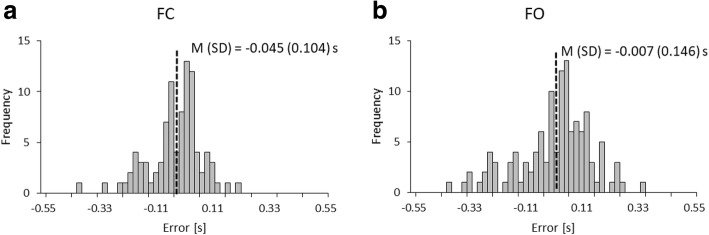
Histogram of frequency distribution of estimation errors: **a**) foot-contact (FC) and **b**) foot-off (FO) time. Mean and standard deviation of the error are represented as M (SD). The x-axis indicates the estimation errors created by subtracting reference system data from the proposed method data (s), and the y-axis indicates frequency of the error

There were no adverse events in the present study. The typical time-course data of leg position calculated from the data of dual laser range sensors is shown in Fig. 2a. The acceleration data was calculated from the second derivative of each leg position and is shown in Fig. 2b. Error distributions for FC and FO are illustrated in Fig. 3. The mean error (Mean ± SD) and variability for FC and FO were small (− 0.045 ± 0.104 s and − 0.007 ± 0.146 s, respectively). In addition, histogram for FC and FO showed monomodality.

**Fig. 4:**
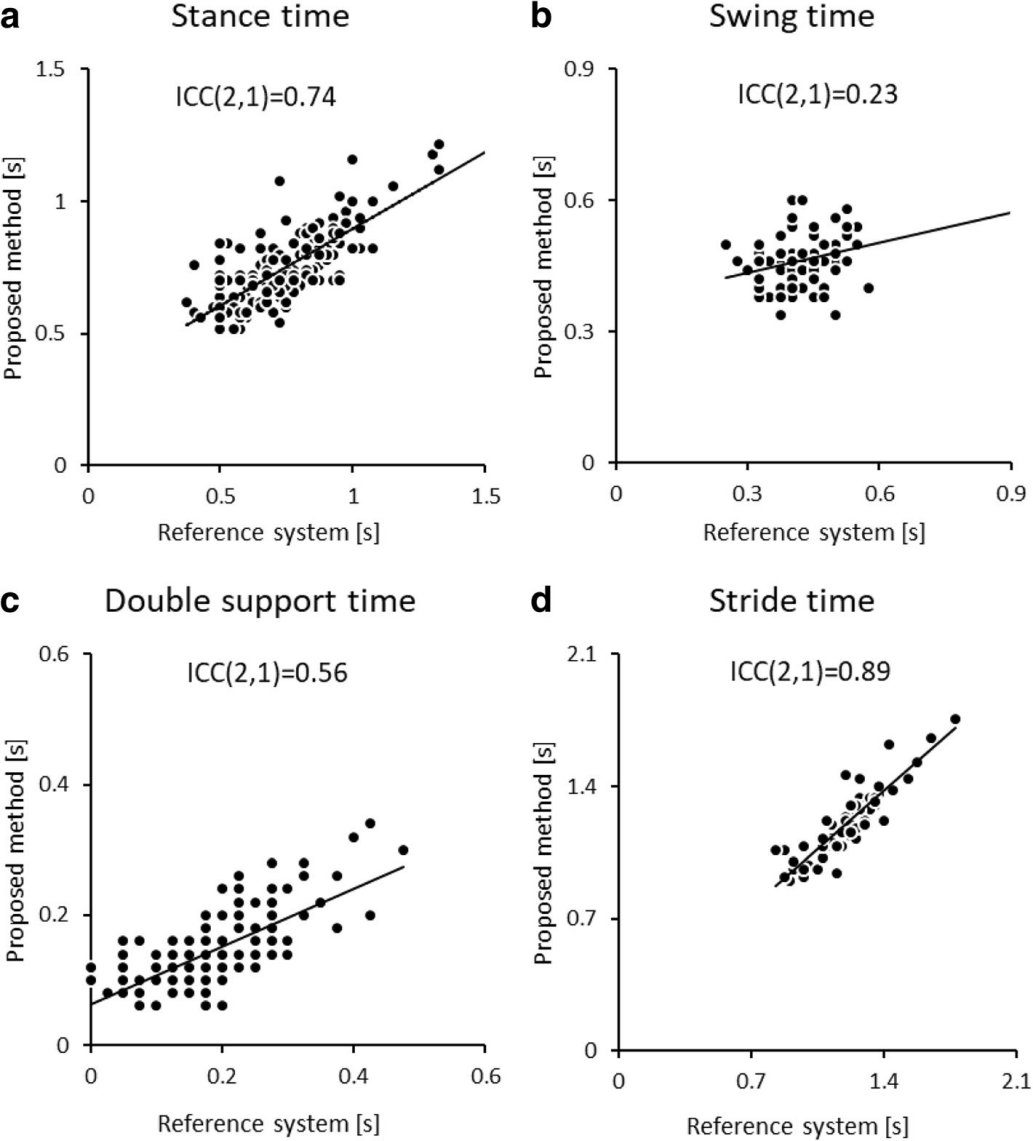
Correlation of temporal parameters obtained from 30 trials (10 participants at three conditions of gait speed) from both systems: **a**) stance time **b**) swing time **c**) double support time and **d**) stride time

**Fig. 5:**
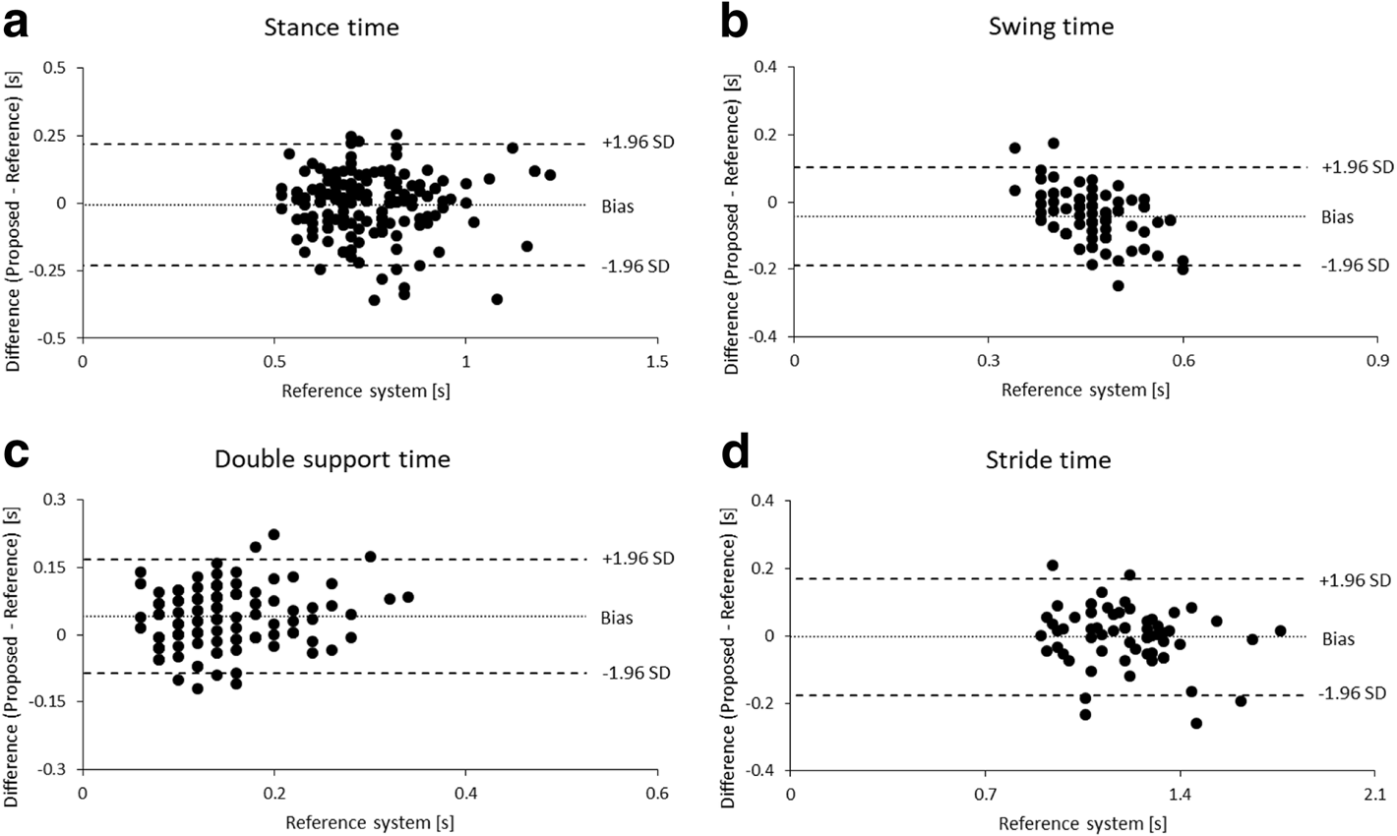
Bland-Altman plot of temporal parameters obtained from 30 trials (10 participants at three conditions of gait speed) from both systems: **a**) stance time **b**) swing time **c**) double support time and **d**) stride time

The summary of spatial and temporal gait parameters is shown in Table 1. The correlations and BA plots of stance, swing, double support, and stride time between two systems are shown in Figs. 4 and 5. The results revealed a good correlation in stride time (0.89). However, the rest of the temporal parameters (stance, double support and swing time) did not reach a good level of correlation (0.74, 0.56 and 0.23, respectively). The systematic errors of each temporal parameter excluding swing and double support time (*P* < 0.001) were not observed (*P* = 0.59–0.81).

**Table 1:** Concurrent validity between proposed and reference system for spatial and temporal gait parameters

	Proposed system mean ± SD	Reference system mean ± SD	Absolute error mean ± SD	Percentage of error (%) mean ± SD	ICC (2,1) [95% CI]	95% LoA
Stance time (s)	0.744 ± 0.181	0.749 ± 0.136	0.087 ± 0.075	11.73 ± 9.92	0.74 [0.67 to 0.80]	−0.23 to 0.22
Swing time (s)	0.420 ± 0.069	0.462 ± 0.055	0.066 ± 0.055	14.24 ± 11.61	0.23 [0.22 to 0.42]	−0.19 to 0.11
Double support time (s)	0.188 ± 0.092	0.146 ± 0.057	0.062 ± 0.045	47.40 ± 38.46	0.56 [0.26 to 0.73]	−0.09 to 0.17
Stride time (s)	1.205 ± 0.192	1.208 ± 0.189	0.064 ± 0.061	5.41 ± 5.19	0.89 [0.83 to 0.94]	−0.18 to 0.17
Stride length (cm)	113.1 ± 18.48	114.04 ± 15.97	7.81 ± 6.34	6.75 ± 5.25	0.83 [0.74 to 0.89]	−20.66 to 18.77
Step length (cm)	57.87 ± 10.48	57.78 ± 8.73	5.60 ± 4.75	9.50 ± 7.59	0.71 [0.60 to 0.79]	−14.33 to 14.51

*SD* standard deviation, *ICC* intraclass correlation coefficient, 95% CI: 95% confidence interval, 95%LoA: 95% limits of agreement

**Fig. 6:**
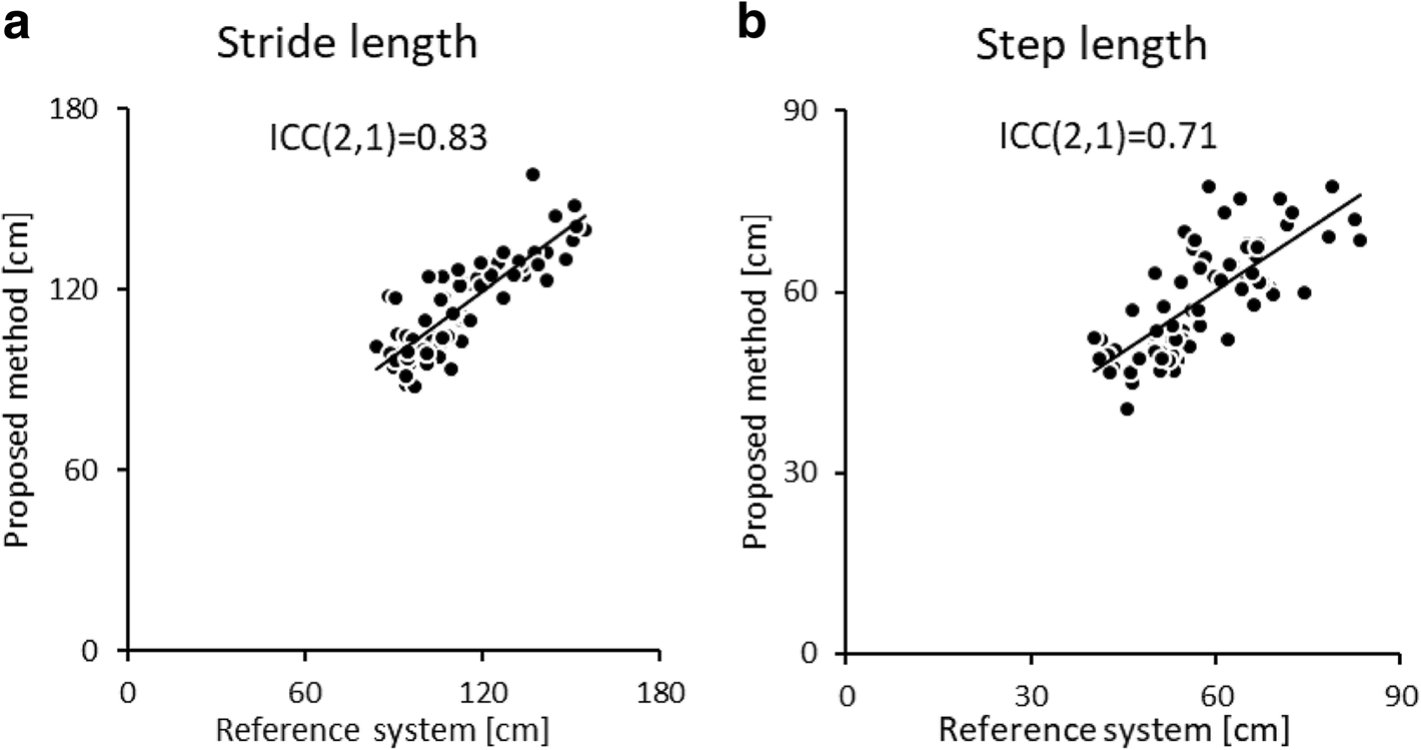
Correlation of spatial parameters obtained from 30 trials (10 participants at three conditions of gait speed) from both systems: **a**) stride length **b**) step length

**Fig. 7:**
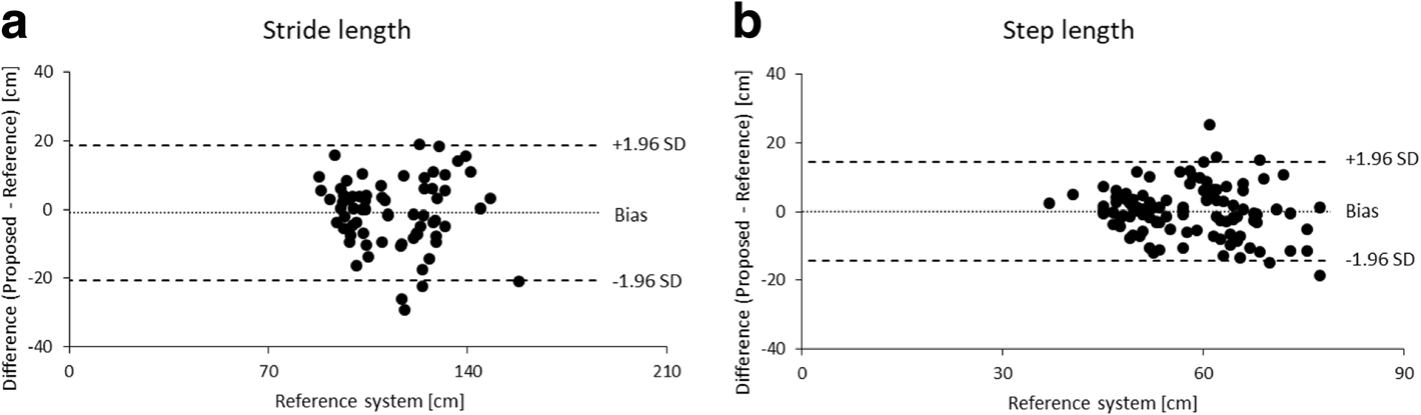
Bland-Altman plot of spatial parameters obtained from 30 trials (10 participants at three conditions of gait speed) from both systems: **a**) stride length **b**) step length

The correlations and BA plots of the spatial stride and step lengths between the two systems are shown in Figs. 6 and 7. The results revealed a good correlation in stride length (0.83), whereas step length did not reach a good level of correlation (0.71). The systematic errors were not observed in each spatial parameter (*P* = 0.43 and 0.90, respectively).

## Discussion

The present study investigated the concurrent validity of the spatiotemporal parameter measurements from the proposed method using dual laser range sensors compared to the computer-based instrumented walkway system using level gait walking in healthy participants. Error distributions for FC and FO indicated small mean error (− 0.045 s and − 0.007 s, respectively) and monomodality and suggest that estimation of FC and FO by the acceleration data is appropriate. The present results showed almost the same degree of error compared to previous studies (0.029–0.041 s and 0.005–0.006 s, respectively) [10]. In addition, the absolute error of all parameters was not so large compared to previous studies [10, 22].

In the previous method using laser range sensor [11], the raw contour of the leg is incomplete because the sensor provides only one-sided information and the number of data points obtained from the sensor changed with distance from the sensor. To address these problems, the proposed method used dual laser range sensors installed at opposite ends of the walking path.

Although the estimation of FC and FO by the acceleration data is appropriate, the ICC values of each parameter was not high enough. It might mean that this proposed method did not have sufficient concurrent validity for spatiotemporal gait measurement. Previous study reported ICC values between the instrumented walkway system and inertial sensor (stance time, ICC(2,1) = 0.84; stride time, ICC(2,1) = 0.98; swing time, ICC(2,1) = 0.89; stride length, ICC(2,1) = 0.92) [10]. Moreover, Portney et al. [19] suggested that the ICC values should be greater than 0.75 for making decisions.

Especially, the correlations in swing time were poor. This might be caused by two factors. One is the narrow range of measurement values. Previous study mentioned the influence of between- participant variance on the ICC value [23]. The ICC is the ratio of true variance (between- participant variance) to true variance plus error. If the true variance is small, the ICC will appear low. Another factor is that low measurement values with a certain amount of measurement error; the proposed method has a similar measurement error regardless of the magnitude of the measured temporal parameters. A drop in the measured value with a constant measurement error might reduce the concurrent validity of the temporal parameters measured by the proposed method.

### Limitations

There are several limitations in the present study. First, in this study, we set the measurement range to 5 m according to the shortest distance in products on the market. Because the laser is irradiated radially, the measurement precision becomes worse with increases in measurement range. In the future, if spatial resolution becomes higher (i.e., the step angle becomes smaller), more long-range measurement using the laser might be possible. Second, the low sampling frequency of the laser range sensor restricts usage in various conditions such as running. Third, in further study, patients with gait disorders and elderly persons should be measured to determine the actual utility of the proposed method for clinical gait analysis using the actual study populations. Fourth, the aim of this study was to investigate only the concurrent validity of the proposed method. To enhance an actual utility of this proposed method, a further study needs to verify whether this method using double laser sensors is significantly more precise than the method using a single laser sensor and the reliability of the proposed method (e.g. test-retest reliability). Since scanning steps of the sensor are 0.25°, there are only three measure points by the single laser sensor condition given that a diameter of shin is 0.07 m and a distance from the sensor to the target (shin) is 5 m. This small number of measure point is forecast to cause a decrease in the precision of the measurement. Lastly, in this study, the ICC value of all spatiotemporal parameters was not high enough (ICC = 0.23–0.89). Previous studies reported that the ICC value of spatiotemporal parameters is higher than or equal to 0.9 [2, 24]. In addition, the proposed method has a certain amount of variability of measurement error in FC and FO. To decrease the variability of measurement error in FC and FO and consequently increase the ICC value of all spatiotemporal parameters, a further study may need to improve the methodology of this proposed system.

## Conclusions

The proposed lacks sufficient concurrent validity for spatiotemporal gait measurement. Although the advantage of the proposed system is the ease of measurement set up; the proposed system does not need to attach sensors and measure calibration values, further improvement of this proposed system seems necessary.

## Abbreviations

95% LoA: 95% limits of agreement
BA: Bland-Altman
FC: Foot contact
FO: Foot off
ICC: Intra-class correlation coefficient
M: Mean
SD: Standard deviation
